# Incorporation of hair follicles in 3D bioprinted models of human skin

**DOI:** 10.1126/sciadv.adg0297

**Published:** 2023-10-13

**Authors:** Carolina Motter Catarino, Desiree Cigaran Schuck, Lexi Dechiario, Pankaj Karande

**Affiliations:** ^1^ Howard P. Isermann Department of Chemical and Biological Engineering, Rensselaer Polytechnic Institute, Troy, NY, USA.; ^2^ Center for Biotechnology and Interdisciplinary Studies, Rensselaer Polytechnic Institute, Troy, NY, USA.; ^3^ Grupo Boticário, Curitiba, Paraná, Brazil.

## Abstract

Current approaches fail to adequately introduce complex adnexal structures such as hair follicles within tissue engineered models of skin. Here, we report on the use of 3D bioprinting to incorporate these structures in engineered skin tissues. Spheroids, induced by printing dermal papilla cells (DPCs) and human umbilical vein cells (HUVECs), were precisely printed within a pregelled dermal layer containing fibroblasts. The resulting tissue developed hair follicle–like structures upon maturation, supported by migration of keratinocytes and melanocytes, and their morphology and composition grossly mimicked that of the native skin tissue. Reconstructed skin models with increased complexity that better mimic native adnexal structures can have a substantial impact on regenerative medicine as grafts and efficacy models to test the safety of chemical compounds.

## Teaser

A physiologically relevant skin model with early hair follicles is developed using 3D bioprinting to improve grafting and testing.

## INTRODUCTION

Human skin comprises three major compartments, the hypodermis, the dermis, and the epidermis, each representing a rich cellular and biomolecular diversity (*1*, *2*). The skin also contains adnexal structures, such as the pilosebaceous unit, which is formed by the hair follicle and sebaceous gland. The pilosebaceous unit is further connected to the sweat apocrine gland, the arrector pili muscle, the underlying vasculature and is in contact with nerve cells (*2*–*4*). This complex structure is formed by about 15 types of cells distributed in concentric layers of cells of epithelial and mesenchymal origins (*3*, *5*). The complexity of this structure confers to this appendage the status of a “mini organ” (*3*, *6*). At early stages of the development of the human body, the interaction between epithelial-mesenchymal cells allows the formation of this skin appendage (*5*, *7*). Through life, different skin stem cell populations support the cyclic regeneration of the hair follicle and sebaceous gland (*5*, *7*). At the base of the hair follicle unit, the dermal papilla region is populated by cells known as dermal papilla cells (DPCs). These cells have a stem cell–like profile that allows the continuous and cyclic regeneration of the hair follicles. This characteristic is also part of the reason why the hair follicle units continue producing fibers in vitro (*8*). Furthermore, besides being an important route of chemical penetration into the skin, the pilosebaceous unit plays a crucial role in wound healing by providing cells that migrate into the damaged area and differentiate into the specific epidermal cells (*9*), demonstrating the relevance of this structure in skin tissue models for both permeation studies and in regenerative medicine as grafts.

Typically, in reconstructed skin models, keratinocytes are directly seeded on top of a dermal scaffold composed of synthetic substrates or matrix derived proteins, such as type I collagen, with or without fibroblasts. Despite their similarity with the native tissue, these in vitro models lack several facets of the complexity (structural, biomolecular, and cellular) of the human skin, such as the presence of the pilosebaceous unit (*10*–*12*). Because of the relevance of the hair shaft for toxicology and regenerative medicine, an evolution of current skin models should focus also on the inclusion of these structures in an attempt to engineer in vitro models or grafts that better mimic the native tissue (*13*).

In the past few years, the study of the hair follicle biology has received notable attention. However, the development of reconstructed skin models containing hair follicles faces critical challenges, such as the loss of the inductive capacity of the human DPCs grown in vitro to regenerate the three-dimensional (3D) structure of the hair follicle and produce hair fiber (*14*, *15*). The “organ germ method” is one of the most successful approaches developed for the regeneration of hair follicle units in vitro and in vivo. In this method, a hydrogel droplet containing self-arranged structures of epithelial and mesenchymal cells forms the “organ germ” (*16*). A common element in these successful studies is the combination of the human cells with mouse cells or the use of only mouse cells and the engraftment of these hair follicle precursors onto animal models (*16*–*18*). Unlike mouse DPCs, human DPCs cultured in monolayers lose their inductive capacity to regenerate the hair follicle, and thus 3D cultures, such as the spheroid model, have been explored as an attempt to overcome the challenges faced with conventional 2D culture (*7*, *19*–*21*). Human DPCs cultured in spheroids have been shown to restore part of their inductive capacity and, once transplanted into human skin grafted onto an animal model, generate hair fibers (*7*, *22*). However, these protocols typically generate the hair follicle units at a small scale and are not always reproducible (*16*, *23*, *24*). Pan *et al.* (*25*) have demonstrated that an array of microwells fabricated by soft lithography, resembling the skin entrances where the pilosebaceous units are found, supported the growth and self-aggregation of DPCs and epidermal cells. However, in their approach, the hair follicles were grown as dissociated units within a gel instead of being part of a reconstructed skin model.

The gaps and limitations in current methodologies have encouraged the search and development of automated approaches, such as 3D bioprinting, that could help facilitate the engineering of complex tissues. 3D bioprinting is an emerging technology that allows the precise placement of materials and cells to create complex structures following specific patterns in a reproducible and high-throughput manner (*22*, *26*). In this proof-of-concept work, we aim to demonstrate the potential of 3D bioprinting to support the fabrication of reproducible hair follicle units from spheroid-based approaches in a high-throughput and reproducible manner and the development of complex reconstructed skin models containing the early hair follicle structures.

## RESULTS

### Viability of 3D bioprinted cells

In this work, we used a pneumatic extrusion-based 3D printing platform. Printing parameters (extrusion pressure, nozzle diameter, and printing velocity) were defined for each individual bioink, based on prior reports and further optimization, to ensure reproducibility of printing (table S1) (*26*–*28*). Using these parameters, we evaluated the viability of bioprinted cells versus manually deposited cells concurrently. Skin cells were evaluated in three groups based on the characteristics of the bioink (viscosity, extent of gelation, and printed volume) in which they are encapsulated and printed in the reconstructed models. Printed human epidermal keratinocytes (HEKs), human epidermal melanocytes (HEMs), DPCs, and human umbilical vein endothelial cells (HUVECs) exhibited high cell viability (greater than 94%), which was comparable to manually deposited cells (figs. S1 and S2).

For the studies on dermal bioink, we generated a set of six hydrogels of type I collagen with human dermal fibroblasts (HDFs) and six hydrogels devoid of cells (negative control) per condition (printed versus manually deposited) for each time point (days 1, 3, and 5). We observed that the metabolic activity and thus the cell viability of the printed versus manually deposited fibroblasts in type I collagen solution were equivalent (fig. S3).

### Characterization of 3D hair follicle spheroid models

**Fig. 1.**
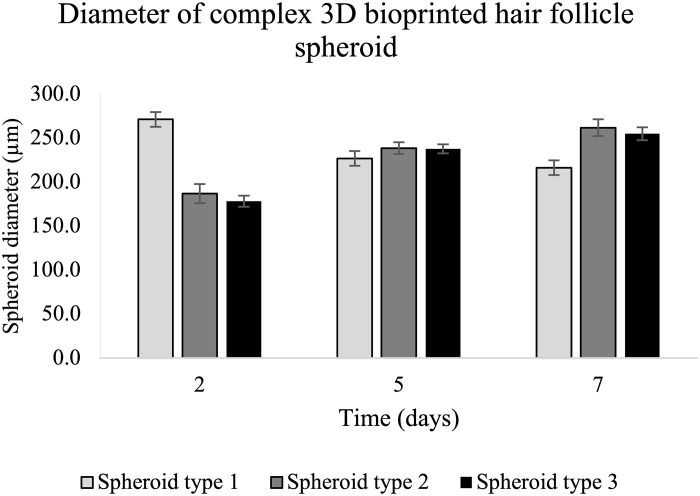
Size of complex 3D bioprinted spheroids generated with different cell compositions. Spheroid type 1: dermal papilla cells (DPCs); spheroid type 2: DPCs, human epidermal keratinocytes (HEKs), and human epidermal melanocytes (HEMs) (10:20:2); and spheroid type 3: DPCs, HEKs, HEMs, and human umbilical vein endothelial cells (HUVECs) (10:20:2:1). The spheroids were imaged at day 2 (24 hours after printing the DPCs and HUVECs), day 5 (24 hours after printing the HEKs and HEMs), and day 7 (last time point of culture). The results represent the average ± SD of the data from a single experiment and *n* = 24 spheroids per condition.

**Fig. 2.**
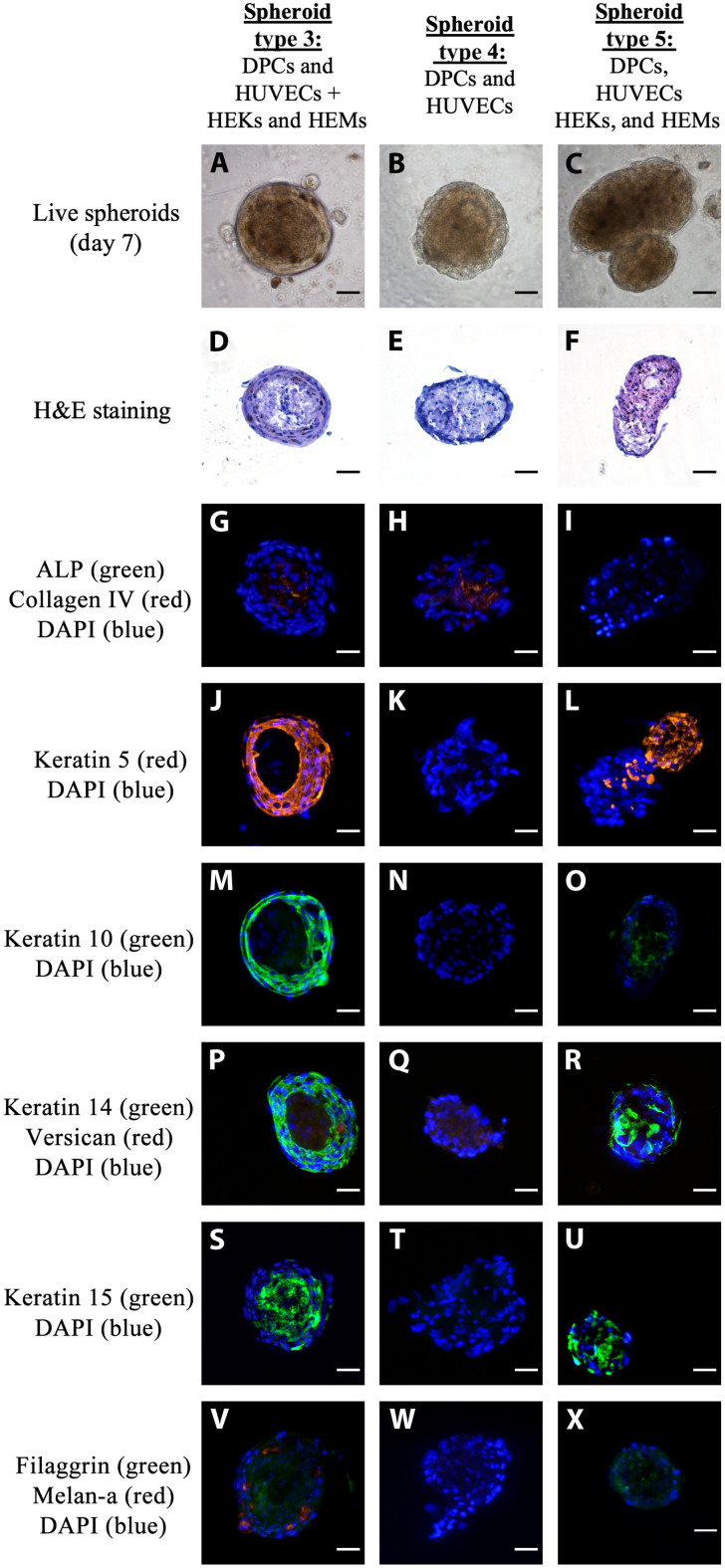
Characterization of complex 3D bioprinted spheroids generated with different cell compositions. Spheroid type 3: dermal papilla cells (DPCs) and human umbilical vein endothelial cells (HUVECs) coprinted at day 1 and human epidermal keratinocytes (HEKs) and human epidermal melanocytes (HEMs) coprinted at day 4 (10:1:20:2); spheroid type 4: DPCs and HUVECs coprinted at day 1 (10:1); and spheroid type 5: DPCs, HUVECs, HEKs, and HEMs coprinted at day 1 (10:1:20:2). (**A** to **C**) Live images of spheroids in culture taken at day 7. (**D** to **F**) Histological analysis: Hematoxylin and eosin (H&E) staining (15-μm sections). (**G** to **X**) Analysis of skin markers by immunofluorescence (15-μm sections). (G to I) Alkaline phosphatase (ALP; green), collagen IV (red), and 4′,6-diamidino-2-phenylindole (DAPI) (blue); (J to L) CK5 (red) and DAPI (blue); (N to O) CK10 (green) and DAPI (blue); (P to R) CK14 (green), versican (red), and DAPI (blue); (S to U) CK15 (green) and DAPI (blue); (V to X) filaggrin (green), melan-a 5 (Red), and DAPI (Blue). Scale bars, 50 μm.

As a first step, we investigated the number of cells that would result in a spheroid with a size similar to the human dermal papilla in vivo (~100 to 250 μm in diameter) (*29*, *30*). Accordingly, spheroids with 3000, 6000, 9000, and 12,000 DPCs each were generated. As seen in fig. S4, the diameter of the spheroids varied from ~250 to ~450 μm and was directly proportional to the initial number of DPCs. We observed that the spheroids condensed over time, forming a dense structure (discussed further in results presented in Figs. 1 and 2).

The spheroids generated with 3000 DPCs were closest in size to that of the human dermal papilla (~100 to 250 μm in diameter). Notably, Higgins *et al.* (*14*), using the same initial number of DPCs, also obtained spheroid of similar diameters (~150 μm). Also, because these dimensions are well below the typical diffusion limit of oxygen and nutrients in nonvascularized tissues, ensuring adequate supply of nutrients for the cells across the spheroids, this cell density was used for all further experiments (*31*).

The interaction between mesenchymal and epithelial cells is a key in the development of the hair follicle structure (*32*–*36*). Accordingly, we included HUVECs, HEKs, and HEMs along with DPCs to form spheroids (fig. S5). Spheroids formed from three different combinations of cells such as type 1 (DPCs only), type 2 [DPCs, HEKs, and HEMs (in the ratio 10:20:2, respectively)], and type 3 [DPCs, HEKs, HEMs, and HUVECs (in the ratio 10:20:2:1, respectively)] were evaluated. Within the first few hours of being seeded in ultralow attachment microplates, the DPCs started to self-assemble and over time resulted in densely packed and well-rounded spheroidal structures. As shown in Fig. 1, the diameter of the spheroids comprising DPCs alone reduced from ~250 μm, 24 hours after seeding, to ~200 μm, at day 7. As expected, the addition of HEKs and HEMs in spheroid types 2 and 3 on day 4 resulted in the enlargement of the aggregates. However, opposed to what was observed in type 1 spheroids, the diameter of spheroids with HEKs and HEMs (types 2 and 3) increased over the time course of the study (days 5 and 7).

We characterized type 3 spheroids comprising DPCs, HUVECs, HEKs, and HEMs for their morphological and biological features using histology and immunofluorescence. We used as control spheroids formed only using DPCs and HUVECs (type 4). Furthermore, to elucidate the relevance of the two-step approach in the formation of well-organized concentric aggregates adopted in the generation of spheroids, we studied an additional spheroid type 5, in which the four cell populations were seeded simultaneously (fig. S6).

Following 7 days in culture, we observed that spheroid types 3 and 4 formed rounded and symmetric spherical aggregates reproducibly (Fig. 2, A and B), similar to intact human dermal papilla (*7*, *14*), while spheroid type 5 resulted in asymmetric aggregates of cells and disperse structures (Fig. 2C). Live and histology images of spheroid types 3 (Fig. 2, A and D) and type 4 (Fig. 2, B and E) revealed morphological resemblances between spheroid type 4 and the core of spheroid type 3. Further, we observed a layer of epidermal cells with distinct morphological characteristics around the core of the spheroid constituted by DPCs and HUVECs in live and in histology images of spheroid type 3 (Fig. 2, A and D). The layer around this core in spheroid type 3 was formed by the epidermal cells printed on day 4. The histological characterization of spheroid type 5 (Fig. 2F) reinforced the lack of symmetry and order of the structure. There was no clear distinction between core and sheath, and the formation of distinct compartments at the upper and bottom region of the cell aggregate was also evident.

In the skin, cytokeratin 14 (CK14) and CK10 are markers of the basal and suprabasal layers of the epidermis, respectively, and also markers of the outer and inner root sheath of the hair follicle, respectively (*7*, *37*). Accordingly, in spheroid type 3, these proteins were restricted to the sheath (Fig. 2, P and M), whereas in spheroid type 5, they were poorly stained throughout the entire structure (Fig. 2, R and O), especially CK10. In spheroid type 4, these proteins were not detected, which is consistent with the absence of HEKs in this model (Fig. 2, Q and N). CK5 is also a marker of HEKs from the outer root sheath of the hair follicle (*32*). As observed in Fig. 2J, CK5 was strongly detected in the sheath of epidermal cells surrounding the core of the spheroid type 3, while it was completely absent in spheroid type 4 (Fig. 2K). In Fig. 2L, once more, the formation of two distinct but interconnected compartments of cells is visible, one of which stained prominently for CK5, while the other lacked its presence.

CK15 is coexpressed with CK5 and CK14 by HEKs from the basal layer of epithelial tissues, and its expression is also linked to hair follicle stem cells (*38*, *39*). In our model, we detected the expression of CK15 in the core of the spheroid type 3 (Fig. 2S) and all over spheroid type 5 (Fig. 2U). This suggests that, in spheroid type 3, the cells in the core of the spheroid, most probably the DPCs, retained some stem cell characteristics. This result is especially important when comparing to spheroid type 4 (Fig. 2T), in which CK15 was completely absent.

We also detected collagen IV in the spheroid types 3 and 4 but not in spheroid type 5 (Fig. 2, G to I), indicating the possible impact of the proper structural organization for the expression of this protein. Filaggrin was also weakly detected only in the samples with epidermal cells (Fig. 2, V and X). This poor expression of filaggrin is consistent with impaired final differentiation of the HEKs, which, in the reconstructed skin models, is promoted by the exposure of the tissue to the air-liquid interface. On the other hand, melan-a, a marker for HEMs, was only detected in spheroid type 3 (Fig. 2V), which could result from the organization of the tissue in concentric layers, mimicking the native hair follicle.

Versican expression has been correlated with the inductive potential of DPCs (*14*). This proteoglycan is found at the dermal sheath surrounding the bulge and in the core of the bulb (*37*). In our model, we detected versican weakly in spheroid types 3 and 4, but not in spheroid type 5 (Fig. 2, P, Q, and R, respectively). One possible explanation for the lack of versican in spheroid type 5 is the asymmetric nature of the structures formed in this model, which could result in the sectioning and staining of a portion containing only the epidermal cells.

The activity of alkaline phosphatase (ALP) has also been associated with the stem cell–like behavior of DPCs (*14*). However, in our spheroid models, this enzymatic activity was not detected, which could indicate the reduction or even depletion of the inductive potential of the DPCs, which warrants further investigation.

### Reconstruction of human skin with hair follicle structures

**Fig. 3.**
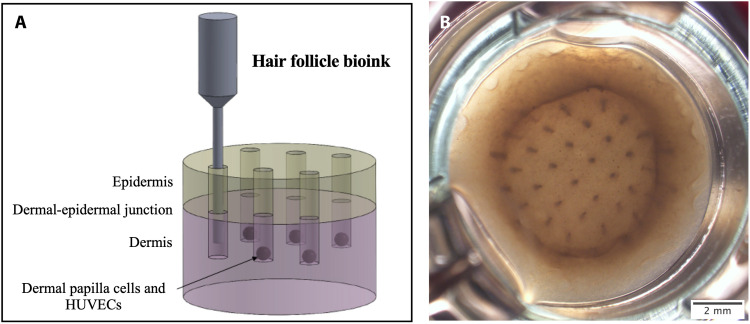
3D bioprinting of hair follicles within skin. (**A**) Schematic of the strategy for printing hair follicle structures within the reconstructed skin models. (**B**) Live image of representative skin model in culture at day 2. Bioinks: Dermal: human dermal fibroblasts (HDFs) resuspended in a solution of rat tail collagen type I; dermal-epidermal junction: collagen IV in cell culture media (2.24 μg per sample); epidermal: human epidermal keratinocytes (HEKs) and human epidermal melanocytes (HEMs) in culture media; hair follicle: mixture of dermal papilla cells (DPCs), HEKs, HEMs, and human umbilical vein endothelial cells (HUVECs) (10:20:2:1). Scale bar, 2 mm.

For the reconstruction of the skin with hair follicle units, we printed the hair follicle bioink comprising DPCs and HUVECs within the crosslinked dermal layer with embedded HDFs (Fig. 3A). A dermal-epidermal junction bioink comprising type IV collagen was printed at the surface of the dermal construct. Following the printing of the epidermal bioink with HEKs and HEMs, we kept the tissue submerged for 3 days and then exposed it to the air-liquid interface for 14 days.

As shown in Fig. 3B, 48 hours after printing the hair follicle bioink in the dermis, we observed the formation of columns containing cells all the way to the surface of the sample. In this specific experiment, the hair follicle bioink was constituted by DPCs, HUVECs, HEKs, and HEMs similar to the composition of spheroid types 3 and 5. The HEMs provided pigmentation to the structures that facilitated the visual observation of the columns. Thus, although the cells were not able to arrange themselves in single aggregates with concentric layers as seen in spheroid type 3, for the initial experiments, we adopted this approach to facilitate the macroscopic observation of the structures.

Results presented above support the utility of bioprinting for creating macroscopic vertical structures in the dermis, thereby suggesting its potential for recreating hair follicles in skin models. Further, it is evident that spheroids generated by combining the DPCs, HUVECs, HEKs, and HEMs in a single step were unable to form an organized structure. In contrast, when we printed epidermal cells after the spheroids of DPCs and HUVECs were already formed, the resulting structure contained cells organized in concentric layers that mimicked their arrangement in the native hair follicle. On the basis of these results, we adopted a two-step approach for the incorporation of hair follicles in the skin model. In this approach, as a first step, we printed DPCs and HUVECs within a preformed dermal layer. We hypothesized that, once printed within the dermis, these cells would form spheroids and subsequently be surrounded by the HEKs and HEMs migrating from the epidermal layer, which is printed on top of the dermal layer in a second step. In addition, on the basis of our previous work on the design of the dermal-epidermal junction bioink (*27*), we speculated that the presence of collagen IV at the interface of the two layers and as a constituent of the hair follicle bioink would promote the migration of epithelial cells toward the spheroids containing DPCs and HUVECs.

**Fig. 4.**
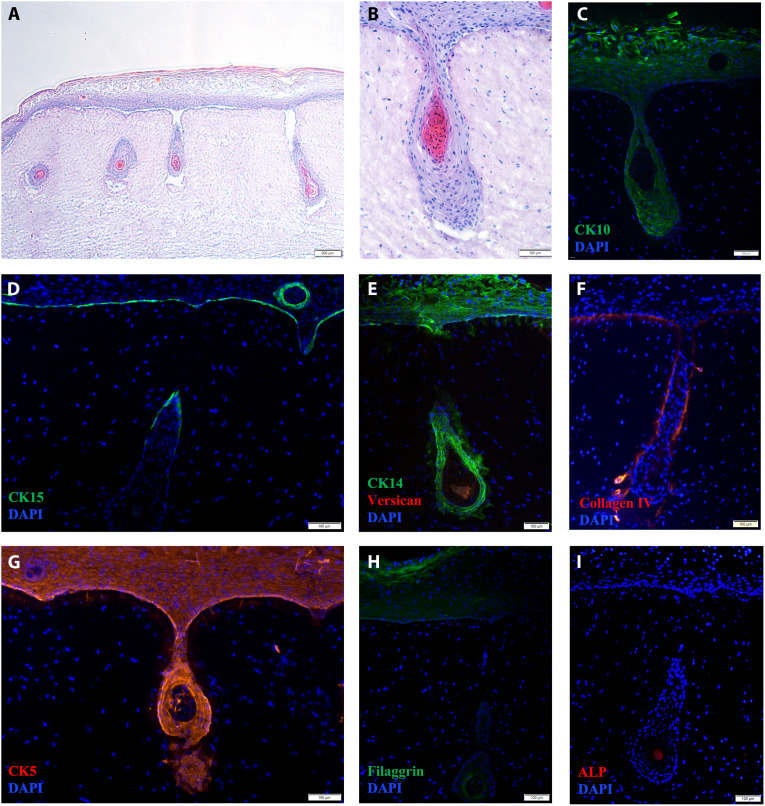
Characterization of 3D bioprinted skin models incorporating hair follicles. (**A** and **B**) Histological analysis. Hematoxylin and eosin stain (15-μm sections). (**C** to **I**) Analysis of skin markers by immunofluorescence (15-μm sections): (C) CK10 (green) and 4′,6-diamidino-2-phenylindole (DAPI) (blue); (D) CK15 (green) and DAPI (blue); (E) CK14 (green), versican (red), and DAPI (blue); (F) collagen IV (red) and DAPI (blue); (G) CK5 (red) and DAPI (blue); (H) filaggrin (green) and DAPI (blue); (I) VECTOR Red alkaline phosphatase (ALP) (red) and DAPI (blue). Scale bars, (A) 200 μm and (B to H) 100 μm.

As shown in Fig. 4, skin models generated using this approach indeed formed a multilayered tissue with a stratified epithelium on top of the dermis. Further, a progressive morphological transition of the HEKs at the stratum basale into corneocytes at the stratum corneum can also be observed (Fig. 4A) (*37*, *40*–*42*). The hair follicle structures presented a morphology that resembled that of the native tissue with outer root sheath and inner root sheath surrounding a dense core (Fig. 4B). Some of these structures were interconnected with the epidermis (follicle like) and others formed spheroids completely surrounded by the dermis (cyst like) (*37*).

The staining of collagen IV (Fig. 4F) highlights the development of a continuous line at the dermal-epidermal junction, which partially contours the hair follicle structure. As previously noted, in the native skin, CK14 and CK10 are found at the basal and suprabasal layers of the epidermis, respectively, and at the outer and inner root sheath of the hair follicle, respectively (*7*, *37*). As seen in Fig. 4 (C and E), we observe that both markers, CK10 and CK14, are uniformly present throughout the entire epidermis. More specifically, CK10 is detected toward the core of the structure, mimicking the inner root sheath (Fig. 4C), while CK14 is found mostly toward the outer region of the hair follicle (Fig. 4E). CK15 is expressed by HEKs at the basal layer of the epidermis and at the upper region of the hair follicle (Fig. 4D). On the other hand, besides being a marker of the outer root sheath of the hair follicle (*32*), CK5 is detected in the HEKs throughout the epidermis and the hair follicle (Fig. 4G), with a stronger expression detected around the core of DPCs and HUVECs.

We detected filaggrin in the upper layers of the epidermis (Fig. 4H), similar to what is seen in the human skin (*40*, *43*, *44*). The protein was also spotted at the inner layer of the epidermal sheath surrounding the spheroids of DPCs and HUVECs, indicating that these epidermal cells are differentiating toward the core of the structure.

As previously noted, versican expression and ALP activity have been associated with the capacity of the DPCs to regenerate the hair follicle (*14*). In our studies, ALP was detected at the central bottom region of the core of the hair follicle (Fig. 4I). On the other hand, as expected, we detected versican in regions closer to the bottom of the hair follicle structure (Fig. 4E).

## DISCUSSION

Although the first reconstructed skin models were developed more than five decades ago, there have been few advances in increasing their complexity, especially with regard to inclusion of multiple representative cells and adnexal structures of the skin (*45*–*47*). None of the current reconstructed skin models available, for example, contain fully developed hair follicle units. The advent of 3D bioprinting technologies can potentially overcome these gaps by facilitating the engineering of niche subcompartments within the gross skin tissue to provide more biologically and physiologically representative skin models for efficacy studies or regenerative medicine. The studies reported here elucidate approaches for addressing these gaps, with particular focus on incorporating hair follicles as an example of skin adnexal structures.

We first assessed the effect of printing processes on cells embedded in different bioinks. Our results demonstrate that the extrusion-based printing approach used in these studies has no effect on the viability of the human primary cells (HEKs, HFNs, HEMs, DPCs, and HUVECs) used in these studies. However, we note that a recent report has demonstrated that although cell viability might not be affected, the printing process can potentially induce inflammatory responses in cells, which can affect tissue functionality and the applications in which 3D printed tissue models are used (*48*).

For the development of spheroids as “organ germs,” we explored different combinations of human primary cell residents within the native hair follicle. In agreement with previous studies, DPCs quickly self-assemble and form spheroids on low-adherence substrates (*7*, *14*, *49*). These aggregates of cells progressively condense, resulting in round shaped and densely packed structures (*14*, *49*). This contraction is likely a combination of the condensation of the structure per se and the absence of proliferation of the DPCs in the spheroid (*14*). To mimic the concentric layers of the hair follicle unit, we included HEKs and HEMs to form the shaft surrounding the core of DPCs and HUVECs. Contrary to what was previously observed in the spheroids with only DPCs or DPCs and HUVECs, when we added the epidermal cells, the diameter of the cell aggregates continued to increase. We speculate that the epidermal cells, similar to what happens in the native skin, continue to proliferate, forming a thicker layer around the dermal papilla core. This two-step approach where the epidermal cells are printed after the DPCs and HUVECs have already formed an aggregate, resulted in a spheroid model that more closely resembles the native human dermal papilla morphologically and biochemically (*7*). The relevance of this approach was highlighted by a lack of uniformity and symmetry in the spheroids with the same cell composition, which had been generated in a single step. These morphological differences have also been observed by Huang *et al.* (*50*). However, the spheroid models in this work did not express some of the expected markers. As noted earlier, versican and ALP activity are commonly used to identify the regenerative potential of DPCs (*14*). In our studies, versican was weakly detected only in spheroid types 3 and 4, and ALP activity was not detected in any of the models. Casale *et al.* (*37*) also have similarly been able to detect versican but not ALP in hair follicle–like structures in their endogenous reconstructed skin model. In the study of Higgins *et al.* (*14*), versican was expressed only in three of the eight cell strains used to generate the spheroids. This indicates that, besides the relevance of the 3D environment to restore the inductive potential of the DPCs, there are also large differences between donor cell populations. Our studies included DPCs from a single donor, and this lack of diversity and representativity could potentially contribute to our observations. Nonetheless, we have demonstrated that 3D bioprinting is a useful platform for the high-throughput fabrication of spheroids for the development of hair follicle units and other organ germ–based approaches.

In vivo, the hair follicle is formed by an elongated structure derived from invaginations of the epidermal layer, in which a hair fiber develops from a mixed population of cells located in the hair bulb. Our strategy to mimic this process was injecting DPCs within a preprinted dermal compartment of the reconstructed skin model (Supplementary Text and fig. S7). This idea is inspired the by 3D bioprinting method known as suspended manufacturing and a technique called FRESH (freeform reversible embedding of suspended hydrogels) (*51*, *52*). In both methods, the bioinks are printed within a “bed” of gel particles of micrometer size or a bath of a uniform thermoreversible gel, respectively. These materials provide support for the fabrication of a complex 3D tissue without relying on the traditional layer-by-layer approach and without interfering with the final structure (*51*, *52*). In the work presented here, instead of printing the hair follicle within a thermoreversible material or a temporary gel bed, we printed it within a collagen gel representing the dermal compartment (Fig. 3A). We hypothesized that we would be able to precisely position the DPCs, along with HUVECs, in a 3D microenvironment, which is known to promote the recovery and maintenance of the hair follicle induction capacity (*7*). Furthermore, with this approach, the displacement of the dermal scaffold by the nozzle creates a channel, which, we speculated, would facilitate the migration of epidermal cells toward the spheroid, recreating the elongated structure of the hair follicle. We observed the formation of macroscopic pigmented verticals channels within the skin (Fig. 3B). However, an unanswered question is if the cells within these openings are arranged randomly or are autonomously organizing themselves and forming a hair follicle structure with all the concentric layers.

Characterization of the printed structures revealed formation of hair follicle–like structures and biomarkers similar to the native tissue. As we hypothesized, the HEKs and HEMs from the epidermal layer migrated toward the spheroid of DPCs and HUVECs through the openings created by the nozzle that printed the hair follicle bioink into the dermis. Similarly, Abaci *et al.* (*32*) have shown that HEKs, seeded on top of a dermal matrix with spheroids formed at the bottom of artificially created vertical channels, migrated toward these cell aggregates, engulfing them and forming a structure that mimicked the native hair follicle. Vahav *et al.* (*30*) have also demonstrated that epidermal cells invaginated toward spheroids of DPCs embedded in a dermal matrix of a reconstructed skin model. These studies emphasize, once more, not only the importance of the interaction between mesenchymal and epithelial cells but also the relevance of the proper 3D and temporal organization, which 3D bioprinting can facilitate.

Our studies reveal the correct arrangement of DPCs into spheroids that are surrounded by HEKs connected to the epidermal layer. Nonetheless, in our model, the hair follicle still lacks a higher degree of differentiation of the epidermal cells surrounding the dermal papilla (*32*, *37*). Possibly, by extending the culture time and optimizing the culture medium, the HEKs can further differentiate into the different hair lineages: outer root sheath (CK5 marker), inner root sheath (AE13, AE15, and CK71 markers), and hair medulla and companion layer (CK71 marker), which remains to be investigated (*15*, *32*, *53*, *54*).

In summary, we have been able to recreate a complex model of the human hair follicle in an automated manner using 3D bioprinting. The approach resulted in a structure with concentric layers of cells that mimics the 3D organization of the human hair follicle. This model could be a useful tool for high-throughput screening of substances for their potential toxicity or regenerative potential toward hair follicle cells. In this work, we have also shown that it is possible to generate a completely 3D bioprinted skin model containing hair follicle structures using only human primary cells. Remarkably, as an improvement on previous efforts on hair follicle regeneration, the 3D bioprinting approach can support scaling up the fabrication of not only dissociated spheroids but also reconstructed skin models incorporating hair follicles with enhanced resolution, speed, and flexibility.

This proof-of-concept work allows us to better understand the potential and applicability of 3D bioprinting technology for the development of other 3D complex structures within the skin, such as vasculature and sweat and sebaceous glands. Last, the approach presented here could substantially affect the way hair follicle models are generated and is likely to affect the field of regenerative medicine as well as safety and efficacy evaluations in the cosmetic and pharmaceutical industries.

## MATERIALS AND METHODS

### Culture of primary cells

HEKs, HEMs, and HDFs were isolated from neonatal foreskin samples donated by D. M. Owens at Columbia University as described previously (*55*–*57*). The samples were obtained under Institutional Review Board protocol AAAB2666. HEKs were cultured in the KGM Gold Bullet Kit medium (Lonza, #192060) supplemented with isoproterenol hydrochloride 10^−6^ M (Sigma-Aldrich, #I6504) and maintained at 37°C and 7.5% CO_2_. HDFs and HEMs were cultured in Dulbecco’s modified Eagle’s medium (DMEM) (Gibco by Life Technologies, #11995) supplemented with 10% fetal bovine serum (FBS) (Atlanta Biologicals, #S115500H) and Medium 254 (Gibco by Life Technologies, #M254500), supplemented with human melanocyte growth supplement (Gibco by Life Technologies, #S0025), respectively. HDFs and HEMs were maintained in a humidified chamber at 37°C containing 5% CO_2_. Cells were subcultured when they reached approximately 70% confluency (treatment with 0.05% trypsin-EDTA, Gibco by Life Technologies, #25300-054). In addition to these cells, for the development of hair follicle 3D models, we also used HUVECs and human hair follicle DPCs. The primary HUVECs were purchased from Lonza (#CC2517A) and cultured in EGM-2 MV Microvascular Endothelial Cell Growth Medium-2 BulletKit (Lonza, #CC-3202). The DPCs were purchased from PromoCell (#C-12071) and cultured in the Follicle Dermal Papilla Cell Growth Medium Kit (PromoCell, #50-306-258). HUVECs and DPCs were also maintained in a humidified chamber at 37°C containing 5% CO_2_ and subcultured by treatment with 0.05% trypsin-EDTA and the PromoCell Detach Kit (#5-–306-390), respectively, when they reached approximately 70% confluency.

### Analysis of viability of printed cells using the BioX 3D bioprinter

To investigate the influence of the printing process on the viability of the cells, epidermal (HEKs and HEMs), dermal (HDFs), and hair follicle (DPCs and HUVECs) cells were printed using the BioX bioprinter and the printing parameter specified for each bioink (table S1). A total of 30,000 epidermal cells (HEKs or HEMs) and “hair follicle” cells (DPCs and HUVECs) were printed in 12-well plates. For the control condition, the same cell density was manually deposited in the wells. The cells were maintained in a humidified chamber at 37°C and 7.5% and 5% CO_2_, respectively, for up to 48 hours with media change every other day. Cell proliferation was quantified by cell counting using trypan blue, allowing the quantification of dead and live cells (*58*). At each day, for each cell type, three wells with printed cells and three wells with manually deposited cells were analyzed. The viability of printed epidermal and hair follicle cells were compared to that of cells manually deposited. For viability analysis of HDFs, 100 μl of a hydrogel composed of rat tail type I collagen, with (1.5 × 10^5^ cells/ml) or without fibroblasts (control), was added (manual deposition) or printed to individual wells of a 96-well plate. The gels were maintained in a humidified chamber at 37°C and 5% CO_2_ for up to 3 days with daily media change. One set of plates was used to perform a metabolic assay at 24, 28, and 72 hours. The gels were manually transferred to a new plate, and a solution of PrestoBlue (Invitrogen, #A13261) in DMEM supplemented with 10% FBS (1:9) was added to the wells. After 5 hours of incubation in PrestoBlue, resazurin (nonfluorescent component of PrestoBlue reagent) is metabolized by live cells and is converted to its fluorescent form. The fluorescence of the supernatant was measured using a plate reader (560/590 nm). Cell viability was calculated by normalizing fluorescence readings of hydrogels containing cells to the controls (hydrogels without cells).

### High-throughput generation of 3D hair follicle spheroid models

Hair follicle models were generated with different compositions of cells (DPCs, HUVECs, HEKs, and HEMs) printed or manually deposited in ultralow attachment 96-well plates. We analyzed the relationship between cell density and spheroid size, the effect of the printing process on the spheroids, and the morphology of the 3D hair follicle models. We analyzed the size of spheroids generated with 3000, 6000, 9000, and 12,000 DPCs. A total of 180 μl of DMEM (Gibco by Life Technologies, #11995) supplemented with 10% FBS (Atlanta Biologicals, #S115500H) was manually added to the wells of the 96-well Spheroid Microplates (Corning, #4515). After trypsinization, the cells were resuspended in DMEM with 10% FBS and centrifuged. The process was repeated a second time to eliminate any trace of trypsin that could affect the formation of the spheroids (*49*). The cells were then resuspended in 20 μl of the same media and manually deposited in each well (ultralow attachment 96-well plates). A total of 10 spheroids were imaged 24 and 48 hours after cell deposition. All images were obtained using the Olympus IX51 Fluorescence Microscope, and the diameter of the spheroids was measured using ImageJ software (ImageJ, U.S. National Institutes of Health, Bethesda, Maryland, USA). The results are presented as the average ± SD.

We further compared spheroids formed by three different combinations of cells: spheroid type 1 (DPCs), spheroid type 2 [DPCs, HEKs, and HEMs (10:20:2)], and spheroid type 3 [DPCs, HEKs, HEMs, and HUVECs (10:20:2:1)]. At day 1, the DPCs (spheroid types 1, 2, and 3) and HUVECs (spheroid type 3) were resuspended in 20 μl of DMEM with 10% FBS and bioprinted in the wells of the ultralow attachment 96-well microplates prefilled with 180 μl of media as previously described. On day 4, 20 μl of media was removed from the wells of spheroid types 2 and 3, and HEKs and HEMs resuspended in 20 μl of DMEM with 10% FBS were printed. The HEKs and HEMs were also centrifuged twice before being printed into the wells of spheroid types 2 and 3. A total of 3000 cells were used for the generation of each spheroid. A 32-gauge nozzle (nominal inner diameter: 0.108 mm) and 35-kPa pressure were used to extrude the bioinks, and the media change was performed by replacing 100 μl of old media with fresh media (fig. S5). For the morphological characterization of the 3D bioprinted hair follicle spheroid models, three combination of cells were tested: spheroid type 3, DPCs and HUVECs coprinted at day 1 and HEKs and HEMs coprinted at day 4 (10:1:20:2); spheroid type 4, DPCs cells and HUVECs coprinted at day 1 (10:1); and spheroid type 5, DPCs, HUVECs, HEKs, and HEMs coprinted at day 1 (10:1:20:2). Briefly, the cells were resuspended in 20 μl of DMEM with 10% FBS and printed in the wells of ultralow attachment 96-well spheroid microplates prefilled with 180 μl of the same media (fig. S6). All cells were centrifuged twice before being printed in the 96-well plates to remove any traces of trypsin. In addition, on the first day, the media was supplemented with collagen IV (0.0112 mg/ml; Sigma-Aldrich, #7521). For the printed cells, a 32-gauge nozzle (nominal inner diameter: 0.108 mm) and 35-kPa pressure were used to extrude the bioinks. The media was changed by replacing 100 μl with fresh media. Following imaging on day 7, the spheroids were embedded in Tissue-Tek O.C.T. (optimal cutting temperature) (Sakura Finitek, #4583), cryopreserved, and then sectioned (15 μm) for histological and immunohistochemical characterization.

### Reconstruction of 3D bioprinted skin models with human hair follicles

The reconstructed skin model incorporating hair follicle structures was generated by first bioprinting the dermal bioink containing 1.5 × 10^5^ HDFs/ml of a solution of rat tail type I collagen (Corning, #354236) and dermatan sulfate (Sigma-Aldrich, #C3788) (9:1, w/w) (fig. S7). A total of 700 μl of the bioink was extruded through a 30-gauge nozzle using 50-kPa pressure onto polyester inserts (12 mm ø, 0.4 μm in pore size) (Corning Transwell, #3460) following a layer-by-layer pattern. On top of the gelled dermal compartment, we bioprinted a layer of collagen IV to mimic the dermal-epidermal junction. A total of 2.24 μg of collagen IV diluted in 60 μl of Dulbecco’s phosphate-buffered saline (DPBS) (^+^Ca/^+^Mg) was printed using a 32-gauge nozzle and 35-kPa pressure. The samples were kept in the incubator (37°C and 5% CO_2_) for 1 hour before printing the hair follicle bioink. The hair follicle bioink was formed by DPCs and HUVECs (10:1) or DPCs, HUVECs, HEKs, and HEMs (10:1:20:2) that were centrifuged twice in fresh media and resuspended in DMEM supplemented with 10% FBS and collagen IV (0.0112 mg/ml). A total of 3000 cells in 0.1-μl volume were extruded within the pregelled dermal layer to form a single hair follicle structure (fig. S7). In each sample, 41 hair follicle units were printed using a 34-gauge nozzle and 25-kPa pressure. To print the hair follicle bioink within the dermis (fig. S7), we used a GCode that is translated by the BioX bioprinter into specific commands to control the movement of the print head, the pressure of the compressor, and the extrusion length of the bioinks. The printer was calibrated to define points *X* = 0, *Y* = 0, and *Z* = 0 (point 000). For the generation of the hair follicle structure in the skin models, the “000” point is set at the center of the sample, right on top of the dermal layer. Figure S7 (bottom right) displays part of the GCode with the list of commands for the generation of 1 of the 41 hair follicle units printed in each sample. Line 2 (T2) defines one of the three printheads containing the bioink, which is also the same printhead used to calibrate the “000” point. The command in line 3 (G1 X-2 Y-4) moves the printhead to the position where the hair follicle bioink is to be printed and varies for each one of the 41 hair units. After the nozzle is at the specific *XY* position, line 5 (G1 Z-1.5) instructs the nozzle to move 1.5 mm bellow the Z0 position. Because the Z0 has been set at the surface of the dermis, this movement will end up placing the nozzle 1.5 mm into the dermal layer. With the next vertical movement (line 8: G1 Z-0.5), the nozzle moves 1 mm higher, leaving a gap into which the bioink is extruded (lines 11 to 13). Once the 0.1 μl of the hair follicle bioink has been extruded (pressure turned on for 0.2536 s), the print head moves up to *Z* = 3 mm (line 14, G1 Z3 F120). This process is repeated until each one of the 41 hair follicle units has been printed. After printing the hair follicle bioink, the epidermal bioink, which is formed by HEKs (2.5 × 10^5^ cells/ml) and HEMs (0.25 × 10^5^ cells/ml) in differentiation medium [KGM Gold BulletKit (1:1) media], is bioprinted on top of the dermal-epidermal junction and dermal layer using a 32-gauge nozzle and 35-kPa pressure. The differentiation medium composition is DMEM and Hams-F12 medium (Gibco by Life Technologies #21700-075) (3:1), 10% FBS and supplements [insulin (5 μg/ml; Sigma-Aldrich, #I2643)], human recombinant epidermal growth factor (10 ng/ml; Sigma-Aldrich, #E9644), 0.1 nM cholera toxin (Sigma-Aldrich, #C8052), transferrin (5 μg/ml; Sigma-Aldrich, #T1147), and hydrocortisone (0.4 μg/ml; Sigma-Aldrich, #H4881). The samples were maintained under submerged condition for 3 days with daily media change [differentiation medium: KGM Gold BulletKit (1:1) media] and then exposed to the air-liquid interface for 14 days. During this period, the tissue was maintained with differentiation medium at the bottom compartment. Following that, the reconstructed skin samples were embedded in Tissue-Tek O.C.T. (Sakura Finitek, #4583), cryopreserved, and then sectioned for histological and immunohistochemical characterization using a HM 505 E cryostat.

### Histological and immunohistochemical characterization of the hair follicle spheroids and reconstructed skin model with hair follicle structures

The samples were morphologically characterized using hematoxylin and eosin (H&E) staining and immunohistochemical staining. Before performing the H&E staining, the frozen sections were maintained at room temperature for at least 4 hours and hydrated for 30 min in water. The sections were stained with hematoxylin for 2 min, washed with tap water for 5 min, and then stained with eosin for 9 min (*59*). The sectioned tissues were sequentially dehydrated in ethanol solution [50, 70, 90, 95, 100 (I), 100 (II), and 100% (III)] and xylol. The slides were mounted with coverslip using DPX mount medium (Sigma-Aldrich, #06522). For the immunofluorescence assay, the slides were also maintained at room temperature for at least 4 hours. The sections were then fixed with cold acetone (−20°C) for 10 min and the tissue was rehydrated with PBS for 15 min. Following that, the fixation sites were saturated by immersion in a solution of PBS and goat serum (10%) and the tissue was permeabilized by briefly immersing it in a solution of Tween 20 in PBS (0.05%). The following antibodies were used for immunolabeling the samples: anti-versican (dilution 1:200; Invitrogen, PA11748A), anti-CK10 (dilution 1:100; Abcam Cambridge, UK, 76318), anti-CK15 (dilution 1:100; Abcam Cambridge, UK, 80522), anti-CK14 (dilution 1:200; Abcam Cambridge, UK. 7800), anti-CK5 (dilution 1:800; BioLegend, USA, 905501), anti-filaggrin (dilution 1:100; Abcam Cambridge, UK, 81468), anti-Ki67 (dilution 1:100; BD, 6100968), and anti–collagen IV (dilution 1:200; Abcam Cambridge, UK, 6311). VECTOR Red Alkaline Phosphatase Substrate Kit was used to characterize the ALP activity of the DPCs. The following secondary antibodies with fluorophores were used for detection: Alexa Fluor 555 (goat) anti-rabbit (dilution 1:200; Abcam Cambridge, UK, 150078), Alexa Fluor 488 (goat) anti-mouse (dilution 1:200; Invitrogen, a11001), Alexa Fluor 555 (goat) anti-mouse (dilution 1:200; Abcam Cambridge, UK, 150114), and Alexa Fluor 488 (goat) anti-rabbit (dilution 1:200; Invitrogen, a11034). The slides were mounted using VECTASHIELD with 4′,6-diamidino-2-phenylindole (nuclear blue stain), and all images were obtained and analyzed using an Olympus IX51 fluorescence microscope.
