# Supplementary material for: Incorporation of hair follicles in 3D bioprinted models of human skin
Source: Sci Adv. 2023 Oct 13;9(41):eadg0297. doi: 10.1126/sciadv.adg0297 (PMC10575578; doi:10.1126/sciadv.adg0297)
Supplement: Supplementary file 1 — Supplementary Text Figs. S1 to S7 Table S1 Legend for movie S1 [file sciadv.adg0297_sm.pdf]

Science Advances
AAAS

Supplementary Materials for

Incorporation of hair follicles in 3D bioprinted models of human skin

Carolina Motter Catarino *et al.*

Corresponding author: Pankaj Karande, karanp@rpi.edu

Sci. Adv. **9**, eadg0297 (2023)
DOI: 10.1126/sciadv.adg0297

The PDF file includes:

Supplementary Text
Figs. S1 to S7
Table S1
Legend for movie S1

Other Supplementary Material for this manuscript includes the following:

Movie S1

Supplementary text

Approach for bioprinting of hair follicles in skin

To print hair follicle structures in the reconstructed skin model, we follow the strategy described in Materials and Methods (***Reconstruction of 3D Bioprinted Skin Models with Human Hair Follicle***) which is also illustrated in Figure S7 and Movie S1. We first print the dermal layer allow it to form a gel, after which we print the hair follicle bioink within the gelled dermis. To achieve this, a nozzle connected to the syringe containing the bioink pierces the dermis until it reaches a specific distance within the tissue and then moves a predetermined distance of 1 mm towards the top surface, leaving a gap into which the hair follicle bioink is extruded. Since the collagen is already crosslinked, the path created by the nozzle remains unsealed. Over time, the cells from the epidermal layer migrate into this channel and produce their own matrix which then promotes cellular interaction and communication between themselves and the surrounding walls of the channel. Thus, using this approach, the thickness of the hair follicle is determined by the growth and development of the cells within the path created by the nozzle. As described in the Discussion section, this approach is inspired by a 3D bioprinting method know as suspended manufacturing and a technique termed FRESH (freeform reversible embedding of suspended hydrogels). The advantage of using 3D bioprinting instead of other approaches such as the use of a pre-patterned mold is that the hair follicle cells are directly deposited into the channel and the "hair density" can be easily programed, with the further possibility of varying density and resolution of the structures when compared to other techniques.

Fig. S1.

Viability of printed and manually deposited human epidermal keratinocytes (HEKs) and human epidermal melanocytes (HEMs). Bioprinting parameters: 32G nozzle and 35 kPa extrusion pressure. The results represent the average ± SD of data from two replicates of n=3.

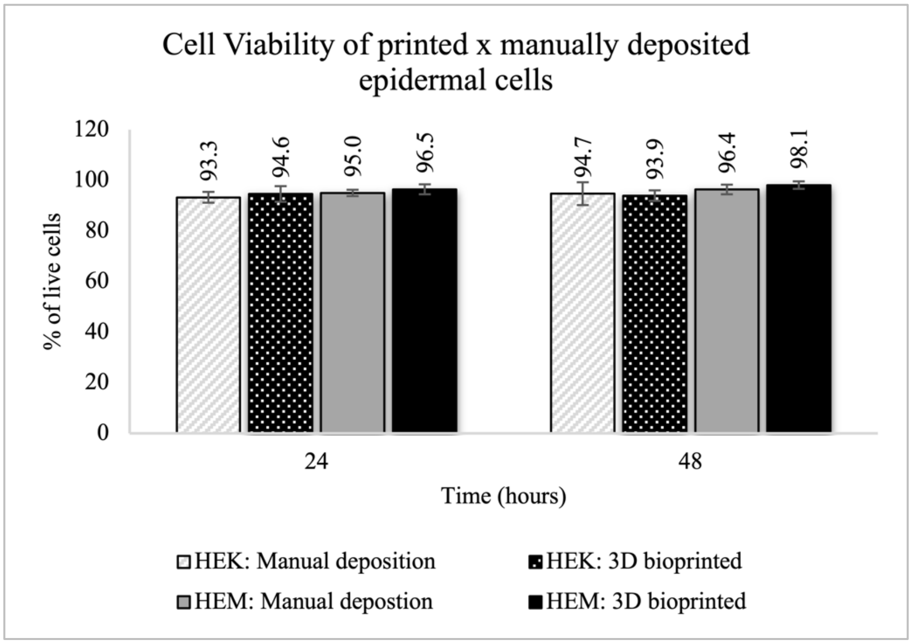

Fig. S2.

Viability of printed and manually deposited human primary dermal papilla cells (DPCs) and human umbilical vein endothelial cells (HUVECs). Bioprinting parameters: 34G nozzle and 25 kPa extrusion pressure. The results represent the average ± SD of the data from two replicates of n=3.

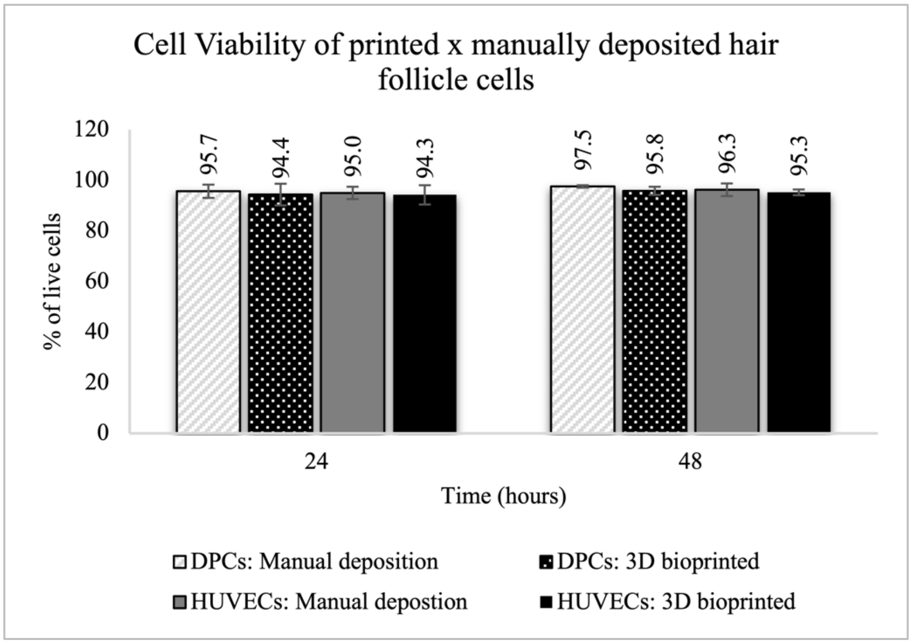

Fig. S3.

Viability of fibroblasts in hydrogels using printing and manual deposition. Bioprinting parameters: 30G nozzle and 50 kPa extrusion pressure. Cell proliferation rate was calculated by normalizing fluorescence readings of hydrogels containing cells to controls of hydrogels without cells. The results represent the average ± SD of the data from two replicates of n=3.

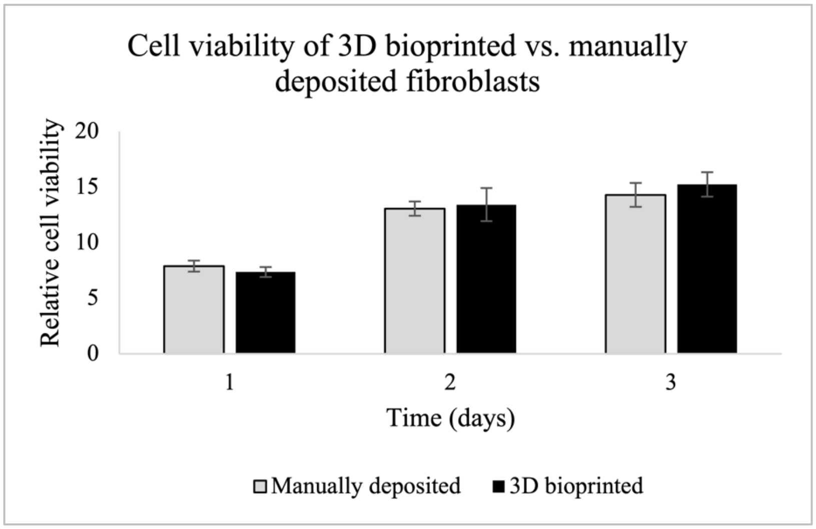

Fig. S4.

Size of spheroids generated manually with different densities of dermal papilla cells (DPCs; 3000, 6000, 9000 and 12000 cells/spheroid). The spheroids were imaged 24 and 48 hours after cell seeding. The results represent the average ± SD of the data from a single experiment (number of spheroids per condition = 10).

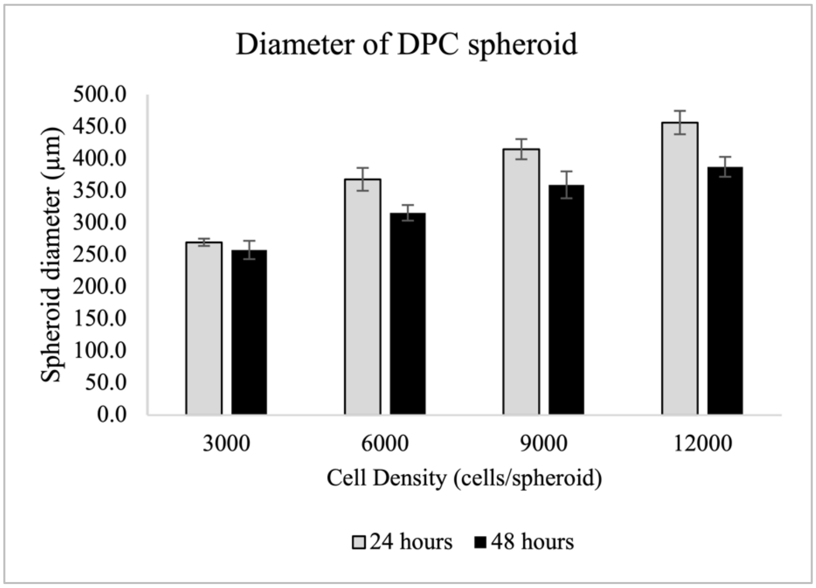

Fig. S5.

Complex 3D bioprinted hair follicle spheroids for size analysis. Scheme for the generation and characterization of spheroids with DPCs, HUVECs, HEKs and HEMs. Numerical values indicate the numbers of each type of cell per spheroid.

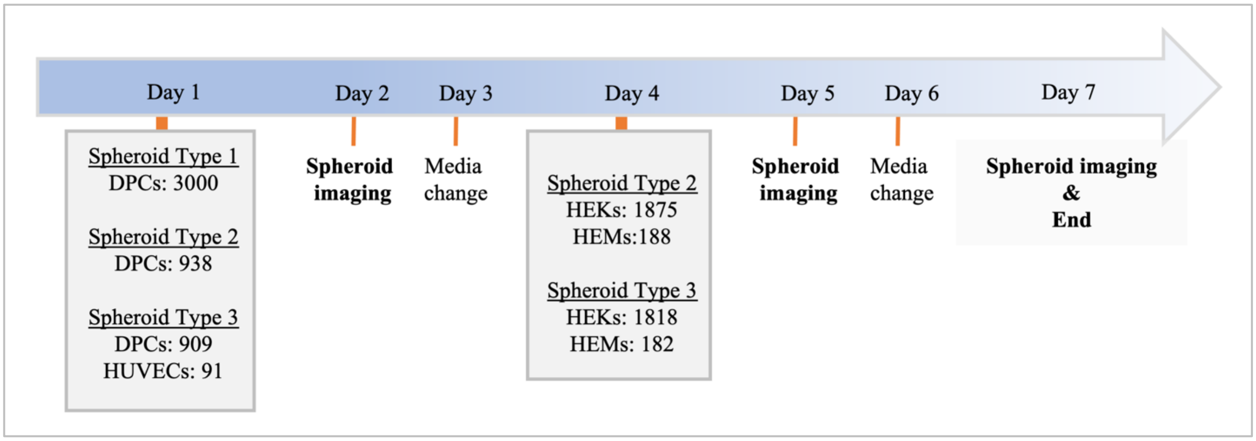

Fig. S6.

Complex 3D bioprinted hair follicle spheroids for histological and immunohistochemical characterization. Scheme for the generation and characterization of spheroids with DPCs, HUVECs, HEKs and HEMs. Numerical values indicate the numbers of each type of cell per spheroid.

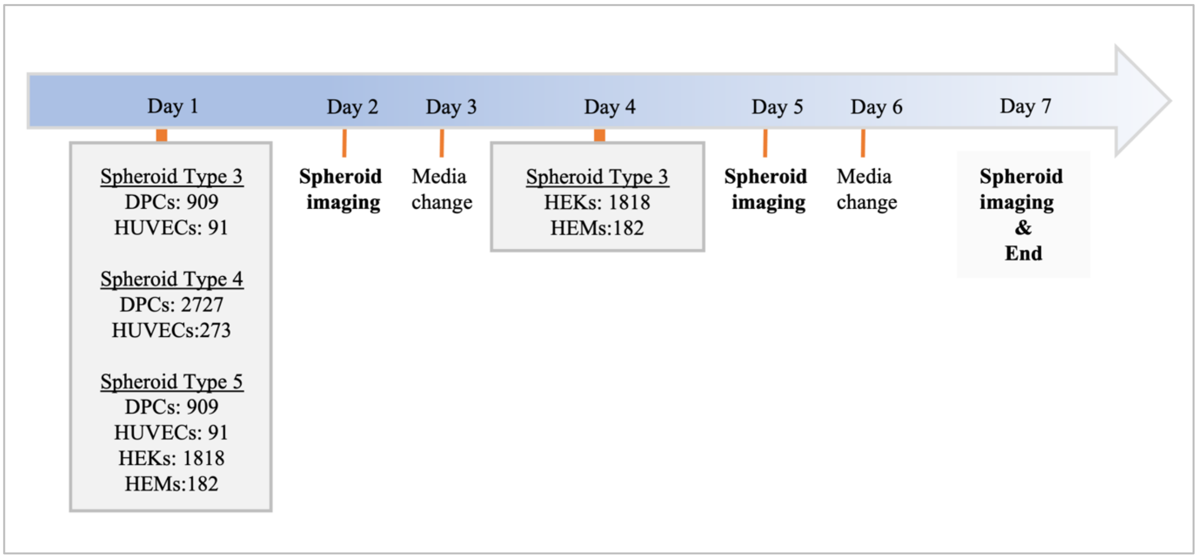

Fig. S7.

Schematic of the strategy for generation of hair follicle structures within the reconstructed skin models. On the bottom right side is presented part of the GCODE used for bioprinting the hair follicle bioink within the dermal layer.

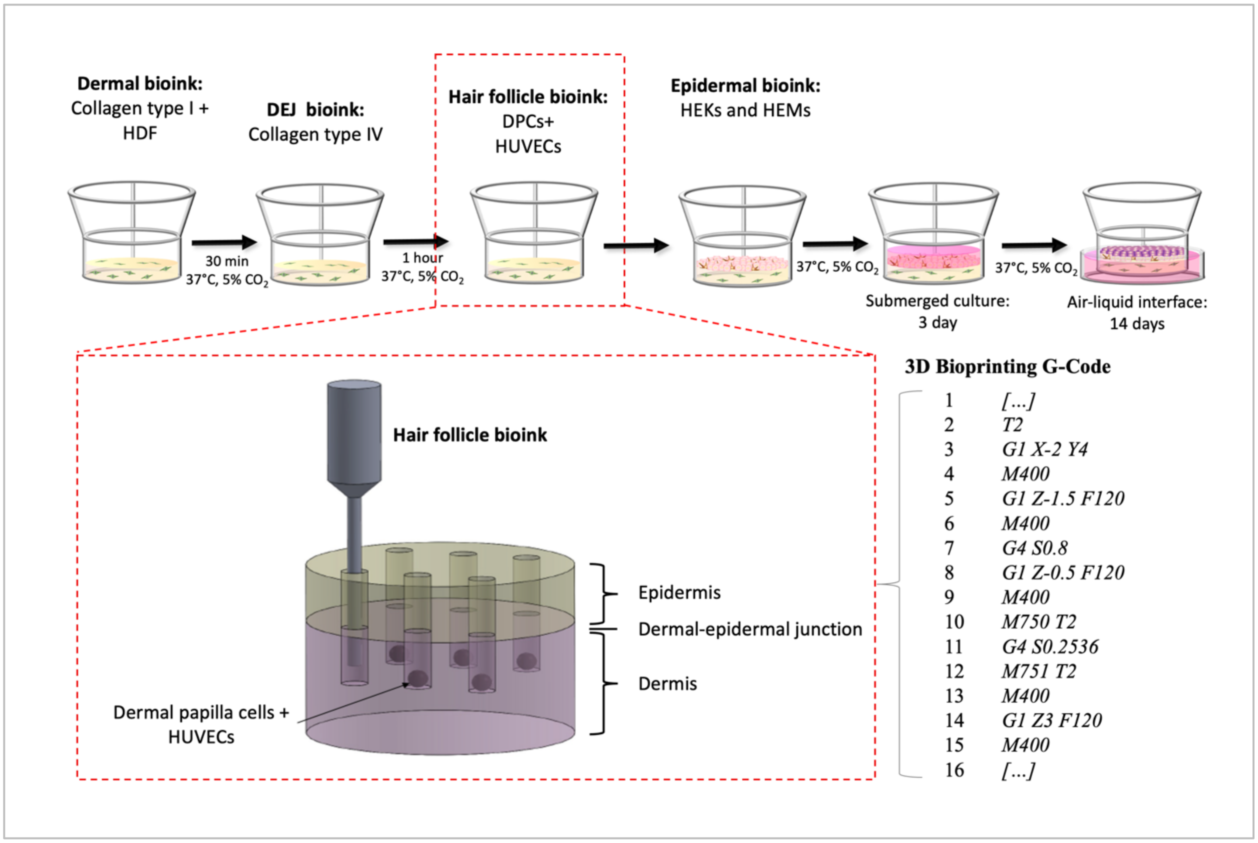

Table S1.

3D bioprinting parameters employed in these studies.

Bioink	Epidermis (1) & Dermal-epidermal junction (2)	Dermis (3)	Hair follicle (4)
**Composition**	Cell culture media	Collagen solution	Cell culture media
**Nozzle diameter (nominal inner ⌀)**	32G (0.108mm)	30G (0.159mm)	34G (0.0826mm)
**Pressure**	35kPa	50kPa	25kPa
**Number of samples**	12	9	12
**Time points**	0.5, 1, 3 and 5 seconds	0.5, 1, 3 and 5 seconds	0.25 and 0.5 seconds

(1) Epidermal bioink: HEKs and HEMs in cell culture media.
(2) Dermal-epidermal junction bioink: collagen type IV solution.
(3) Dermal bioink: HDFs in collagen solution
(4) Hair follicle bioink: DPCs and HUVECs in cell culture media.

Movie S1.
Movie demonstrating the strategy for generation of the hair follicle structure within the reconstructed skin models.

See attached multimedia file.
